# Virtual reality-based training in patients with alzheimer's disease: A systematic review and meta-analysis

**DOI:** 10.1016/j.tjpad.2026.100590

**Published:** 2026-05-05

**Authors:** Junjie Wang, Can Wu, Kedong Zhu, Xiaoshan Qi, Guiqin Chen

**Affiliations:** a School of Physical Education, China University of Geosciences, Wuhan 430074, Hubei Province, China; b Department of Neurology, Renmin Hospital of Wuhan University, Wuhan 430060, Hubei Province, China

**Keywords:** Virtual reality-based training, Alzheimer's disease, Meta-analysis, Cognitive impairment, Systematic review

## Abstract

**Background and Objectives:**

Prior meta-analyses have suggested that training utilizing virtual reality (VR) serves as a secure and effective intervention for elderly individuals experiencing mild cognitive impairment (MCI). Nevertheless, the effectiveness of such interventions appears to differ among various populations and cognitive domains. Furthermore, there remains a significant gap in understanding the effectiveness of VR-based training, specifically among individuals diagnosed with Alzheimer's disease (AD).

**Methods:**

The researchers conducted a comprehensive search of databases, including Web of Science, PubMed, Cochrane Library, and EMBASE up until July 1, 2025, focusing on randomized controlled trials that investigated VR-based training in patients diagnosed with AD. The outcomes measured were categorized and analyzed separately, encompassing overall cognitive performance, distinct cognitive domains, psychosocial function, physical capabilities, and the execution of daily living activities within the context of AD trials.

**Results:**

Of the 265 publications identified, 11 (4.15%) randomized controlled trials (RCTs) eventually met all eligibility criteria. Those who received VR-based training showed significantly better global cognitive function [SMD (95%CI) = 0.44 (0.21–0.68)] and Short-term memory [SMD (95%CI) = 0.62 (0.25–0.99)] than the controls. However, no significant improvements were observed in areas such as executive function, spatial memory, activities of daily living, quality of life, balance and coordination, fear of falling, risk of falls, and depression levels.

**Conclusion:**

VR-based interventions demonstrated beneficial effects on global cognitive function and short-term memory in AD populations. Due to the small sample size, the current research on evidence for efficacy in people with AD is weak and limited in many indicators.

## 1 Introduction

Alzheimer's disease (AD), the most common neurodegenerative disorder worldwide, is the primary cause of dementia, accounting for an estimated 60–70% of cases. Current global estimates indicate that 57 million people live with dementia, with 10 million new cases diagnosed annually [1]. The prevalence of AD continues to rise steadily, imposing multidimensional burdens on caregivers and public health systems while severely compromising the quality of life for affected individuals [2,3].

Although significant advances have been made in AD research in recent years, the pathogenesis of the disease remains incompletely understood. Effective strategies for early prevention, disease modification, and treatment remain limited. Currently, clinical management largely relies on symptomatic pharmacological treatments [4]. Although novel anti-Aβ antibodies can partially delay cognitive decline in early-stage Alzheimer's disease, their use is confined to this initial phase and is linked to adverse effects, including cerebral edema (ARIA-E) and hemorrhage (ARIA-H). These risks are significantly elevated in APOE4 carriers, further narrowing the therapeutic window [5].

In light of these therapeutic challenges, a growing body of research has investigated non-pharmacological interventions. Several studies have demonstrated that physical exercise [[6], [7], [8], [9]] and cognitive training [10,11] may reduce the risk of AD and slow disease progression. Nevertheless, traditional exercise and cognitive interventions often face issues of poor patient adherence in the AD population [12], and their therapeutic benefits remain controversial [13,14].

Virtual reality (VR)-based training has emerged as a promising clinical tool in neurorehabilitation and offers a novel alternative to conventional exercise and cognitive training approaches [15,16]. By incorporating immersive and multi-sensory spatial environments, VR-based interventions may enhance engagement, improve enjoyment, support decision-making, and reduce negative affective states in older adults [17,18]. A recent meta-analysis indicates that VR-based training can effectively enhance memory, attention, information processing speed, and executive function in older adults with mild cognitive impairment (MCI) [19]. Another systematic review pointed out that although most of the included studies had issues such as small sample sizes, unclear blinding methods, and moderate study quality, it is certain that immersive VR has potential as an effective tool for cognitive training in older adults [20]. However, existing systematic reviews and meta-analyses on VR training have predominantly focused on individuals with MCI [21,22], often without distinguishing between etiological subtypes of MCI (e.g., vascular, frontotemporal, or AD-related). This heterogeneity may compromise the validity and stability of estimated intervention effects.

To specifically evaluate the therapeutic potential of VR-based training in AD patients, this systematic review and meta-analysis exclusively includes participants with AD-related cognitive impairment, ranging from MCI to dementia. Our objective is to determine whether VR-based training can serve as an effective intervention for this patient population.

## 2 Methods

### 2.1 Methodological and reporting standards, registrations

This systematic review and meta-analysis adhered to the methodological standards outlined in the Cochrane Handbook [23] and followed the Preferred Reporting Items for Systematic Reviews and Meta-Analyses 2020 statement (PRISMA 2020) [24]. The review protocol was registered in PROSPERO under registration number CRD420251123470.

Outcomes of interest encompassed ten domains: global cognitive function, executive function, short-term memory, spatial memory, activities of daily living, quality of life, balance and coordination, fear of falling, risk of falling, and depression.

### 2.2 Literature search strategy

A systematic literature search was performed using the electronic databases Web of Science, PubMed, Cochrane Library, and EMBASE from their inception until July 1, 2025. The search strategy incorporated the following key concepts: (Alzheimer* Disease* OR Alzheimer Syndrome OR Alzheimer-Type Dementia OR Alzheimer Type Dementia OR Alzheimer Dementia OR Senile Dementia OR Alzheimer Type Senile Dementia OR Primary Senile Degenerative Dementia OR Alzheimer Sclerosis OR Presenile Dementia OR Acute Confusional Senile Dementia OR Early Onset Alzheimer Disease OR Presenile Alzheimer Dementia OR Late Onset Alzheimer Disease OR Focal Onset Alzheimer's Disease OR Familial Alzheimer Disease*) AND (Exergam* OR Virtual Reality Exercise* OR Active-Video Gam* OR Active-Video Gam* OR video gam* OR VR OR Immersive VR OR virtual-reality intervention OR VR-based intervention OR VR exercise OR VR exercise OR Wii OR Kinect OR Xbox OR Computerized cognitive training) AND (Randomized Controlled trial OR Randomized OR Placebo). We also manually searched the bibliographies of review articles and citations within the included studies.

### 2.3 Inclusion and exclusion criteria

Studies were included if they met the following criteria: (i) enrolled participants with a clinical diagnosis of AD; (ii) evaluated any form of VR intervention, such as VR-based exergaming, video gaming or computerized training and compared with a non-VR intervention as control; (iii) reported pre- and post-intervention changes in cognitive scores or at least one outcome related to the domains of global cognition, memory, fear/risk of falling, balance/coordination, quality of life, activities of daily living, executive function, or depression; (iv) designed as RCTs.

Studies were excluded if they met the following criteria: (i) involved participants with MCI or dementia due to non-AD etiologies (e.g., vascular dementia, frontotemporal dementia, Parkinson’s disease, stroke, diabetes, or psychiatric disorders); (ii) evaluated non-VR interventions (e.g., pharmacotherapy, conventional exercise, or standard cognitive training); (iii) were review articles, protocols, guidelines, conference abstracts, duplicate publications, or had incomplete outcome data.

### 2.4 Data extraction

Two independent investigators (J. W and C. W) independently performed the literature search and data extraction, and abstracted the data into a data extraction form. Extracted information included: publication year, recruitment setting, sample size, mean age, proportion of male participants, intervention details, control type, number of sessions, cognitive test scores, and disease severity. For scales with inconsistent scoring directions, scores were harmonized during extraction and synthesis. Discrepancies were resolved through discussion or by a third reviewer (G.C).

### 2.5 Outcome measures and intervention grouping

Global cognitive function was measured by any type of multi-domain cognitive tests, such as the Mini-Mental State Examination (MMSE) and Alzheimer's Disease Assessment Scale-Cognitive Subscale (ADAS-Cog). Memory was assessed by any type of test, such as the Digit Span Test and the Rey-Osterrieth Complex Figure Test (RCFT). Balance and coordination ability was assessed by any type of test, such as Berg Balance Scale (BBS) and Tinetti Gait and Balance Test (TGBT). Quality of Life and living ability were assessed by any type of test, such as Instrumental Activities of Daily Living (IADL) and Quality of Life-AD (QOL-AD). Depressive state was assessed by Cornell Scale for Depression in Dementia (CSDD). Executive function was assessed by any type of test, such as Frontal Assessment Battery (FAB) and Brixton test. If multiple tools were used within the same domain, the most frequently reported scale across studies was selected for meta-analysis to minimize heterogeneity.

VR-based interventions were categorized into two modalities: VR-exercise interventions and VR-cognition interventions. Interventions were stratified by dose: < 24 sessions and ≥ 24 sessions. Participants were grouped by dementia severity: mild dementia (20 < MMSE ≤ 24) and moderate dementia (10 < MMSE ≤ 20). Control groups comprised three comparators: traditional exercise, traditional cognitive practice, and routine medication therapy.

### 2.6 Data analysis

We synthesized the data through integrated narrative and statistical approaches, utilizing textual summaries and tabular presentations to characterize the key attributes of the included studies, encompassing metadata, population profiles, interventions, controls, and outcome metrics. Experimental results were systematically compiled, and when sufficient data (mean ± SD) were available from ≥ 2 studies within the same comparator group, meta-analyses were conducted using R software (version 4.5.0).

Given continuous outcomes and inconsistent assessment tools across trials, standardized mean differences (SMD; Hedges' g) were calculated to estimate overall and subgroup effects. Analysis encompassed overall cognitive outcomes alongside each cognitive or behavioral domain individually. When studies provided more than one outcome per domain for analysis, we adopted the most commonly used scale data in this domain, or the scale data mostly used in the included studies, thereby ensuring that the heterogeneity of the measurement is minimized as much as possible. Finally, standardized mean differences (SMDs) for VR and control arms were computed per study, then synthesized across trials to both isolate control group nonspecific effects and assess if VR-based training yields authentic cognitive enhancement.

In both overall and subgroup analysis, heterogeneity among studies was assessed using the I² and a chi-square P value. Significant heterogeneity was indicated when the chi-square P value was ≤ 0.05. Study heterogeneity was also quantified via the I² statistic, with values of 25%, 50%, and 75% corresponding to low, moderate, and high heterogeneity, respectively [25]. For I² > 50%, a random-effects model was applied; otherwise, a fixed-effects model was implemented. Sensitivity analysis was performed on pooled effect estimates by sequentially excluding studies that contributed disproportionately to overall heterogeneity.

## 3 Results

### 3.1 Study selection

**Fig. 1:**
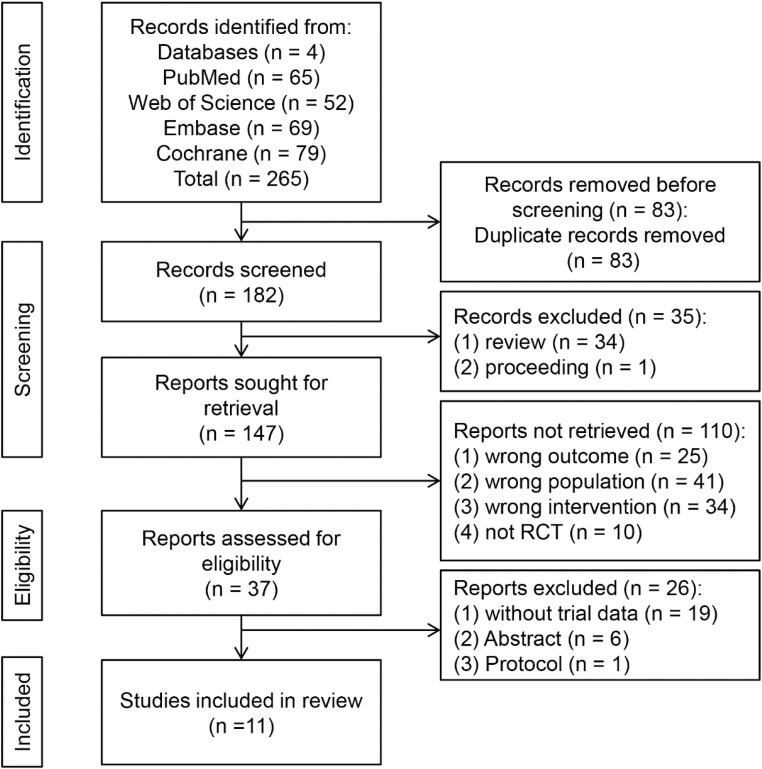
Flowchart of the study selection process.

As delineated in Fig. 1, our systematic search strategy initially identified 265 records. Following duplicate removal (automatically via EndNote X9; manually verified), 182 unique publications remained. Subsequently, 35 publications were excluded as non-empirical articles (reviews, commentaries) or conference proceedings. After screening titles/abstracts against predefined PICOS criteria, 110 irrelevant records were removed, yielding 37 potentially eligible full-text articles. Among these, 19 studies were excluded due to ongoing trials without analyzable data; 6 conference abstracts lacked sufficient data; 1 protocol paper described planned work. Consequently, 11 studies satisfied all inclusion criteria for quantitative synthesis [[26], [27], [28], [29], [30], [31], [32], [33], [34], [35], [36]] (Fig. 1).

### 3.2 Risk of bias

The potential risk of bias for each included study was evaluated using the revised Cochrane Risk of Bias tool for Randomized Trials (RoB-2) [37]. This tool systematically assesses six critical domains of bias: (i) Bias arising from the randomization process; (ii) Bias due to deviations from intended interventions; (iii) Bias due to missing outcome data; (iv) Bias in outcome measurement; (v) Bias in selective result reporting; (vi) Overall risk-of-bias judgment.

**Fig. 2:**
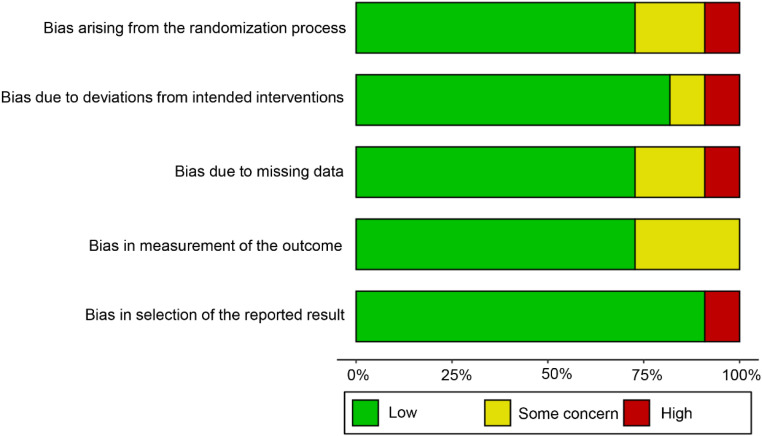
Review authors’ judgments about each “risk of bias” domain.

**Fig. A1:**
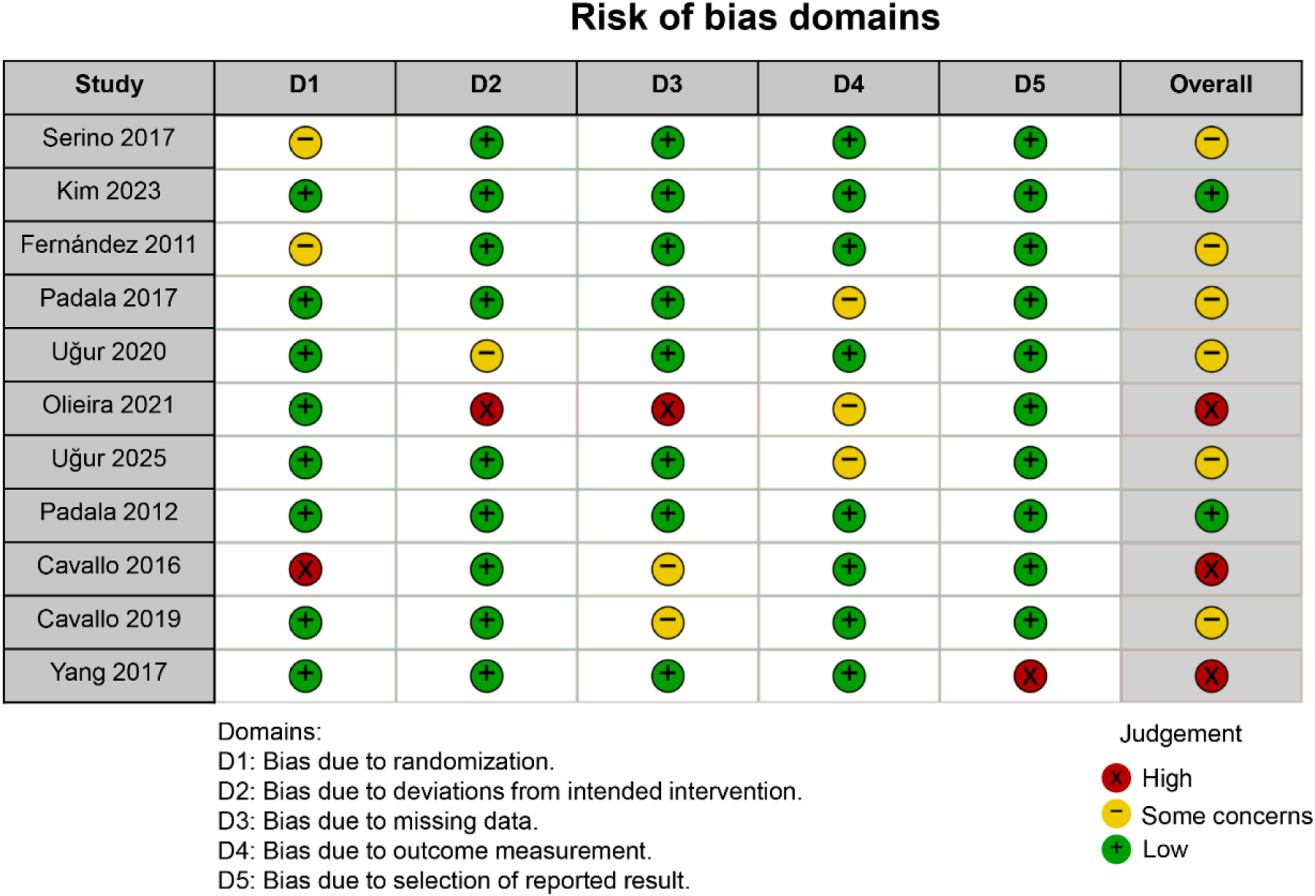
Review authors’ judgments about “risk of bias” domain in each included study.

As shown in Fig. 2, a total of 73% (8/11) of the studies were judged to have a low risk of bias in the “randomization process” domain. With regard to the “deviations from the intended interventions” domain, there was a low risk of bias in 82% (9/11) of the studies. The risk of bias because of missing outcome data was low in 73% (8/11) of the studies. 73% (8/11) of studies were judged to have a low risk of bias in the “measuring the outcome” domain. 91% included studies (10/11) were rated as low risk in the “selection of the reported results” domain. Reviewers’ judgments about each “risk of bias” domain for each included study are presented in Appendix Fig. A1. Assessment was conducted by two independent reviewers (J.W and K.Z). A senior reviewer (G.C) established consensus scores and resolved disagreements.

### 3.3 Characteristics of included studies

The 11 included studies, published between 2012 and 2025, were conducted in six countries: the United States, Italy, Turkey, Portugal, Brazil, and South Korea (Table 1). The mean age of participants ranged from 73 to 87 years, with the proportion of male participants varying from 15% to 72%.

**Table 1:** Characteristics of included studies (N=11).

Study (first author and year)	Country/Region	Mean age	Sex (Male %)	No. of participants (Intervention/Control)	Disease severity	Type of Intervention	Type of control	Training duration	No. of sessions
Serino 2017	Italy	87.65±5.01	15	10/10	Mild dementia	VR Cognitive	Traditional Cognitive	4 weeks	10
Padala 2017	USA	73.0±6.2	63	12/12	Mild dementia	VR Exercise	Walking	8 weeks	40
Uğur 2020	Turkey	73.44±4.36	72	16/16	Moderate dementia	VR Exercise	Routine Medical	6 weeks	12
Padala 2012	USA	80.45±7.75	27	11/11	Mild dementia	VR Exercise	Walking	8 weeks	40
Oliveira 2021	Portugal	83.24±5.66	29	10/7	Moderate dementia	VR Exercise	Routine Medical	6 weeks	12
Fernández 2011	Brasil	-	-	15/15	Moderate dementia	VR Cognitive	Traditional Cognitive	12 weeks	36
Cavallo 2016	Italy	76.42±3.37	38	38/38	Mild dementia	VR Cognitive	Traditional Cognitive	12 weeks	36
Yang 2017	Korea	70.5±7.67	70	10/10	Mild dementia	VR Cognitive	Routine Medical	12 weeks	24
Kim 2023	USA	82.16±8.4	19	8/8	Moderate dementia	VR Cognitive	Traditional Cognitive	5 weeks	10
Uğur 2025	Turkey	73.44±4.36	72	16/16	Moderate dementia	VR Exercise	Routine Medical	6 weeks	12
Cavallo 2019	Italy	-	-	36/36	Mild dementia	VR Cognitive	Traditional Cognitive	12 weeks	36

Note: “-” indicates that the data were not reported in the original studies.

A total of 361 subjects were included, with 182 assigned to intervention groups and 179 to control groups. Six studies enrolled exclusively patients with mild dementia, while five focused on moderate dementia; no studies involved participants with MCI.

The intervention group received VR-based physical exercise or cognitive training, whereas controls received either traditional cognitive training, walking therapy, or routine medical treatment. Intervention duration ranged from 4 to 12 weeks, with the number of sessions varying between 10 and 40. The median session was 24, establishing < 24 sessions and ≥ 24 sessions as the cut-off in the subgroup analysis. Session frequency ranged from 2 to 5 times per week, with each session lasting between 20 and 45 minutes.

Regarding recruitment settings, five studies enrolled participants from social senior centers, rehabilitation centers, or assisted living facilities; two studies recruited from hospitals; the remaining three studies did not specify recruitment locations. Three studies were assessed as high risk, two studies were assessed as low risk, and the remaining studies were assessed as some concerns of bias in the total of the RoB-2 domains.

### 3.4 Global cognitive function in people with AD

**Fig. 3:**
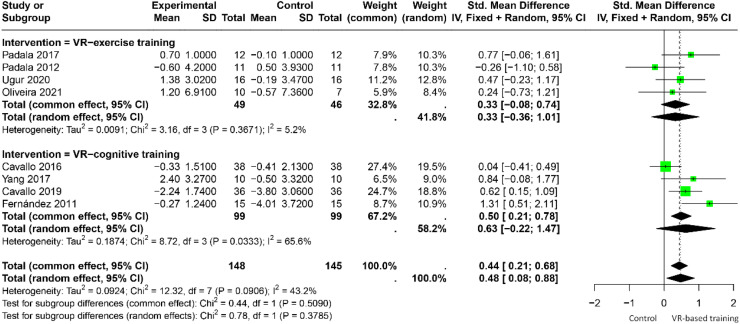
Effect of VR-based training on global cognitive function in patients with AD.

**Fig. A2:**
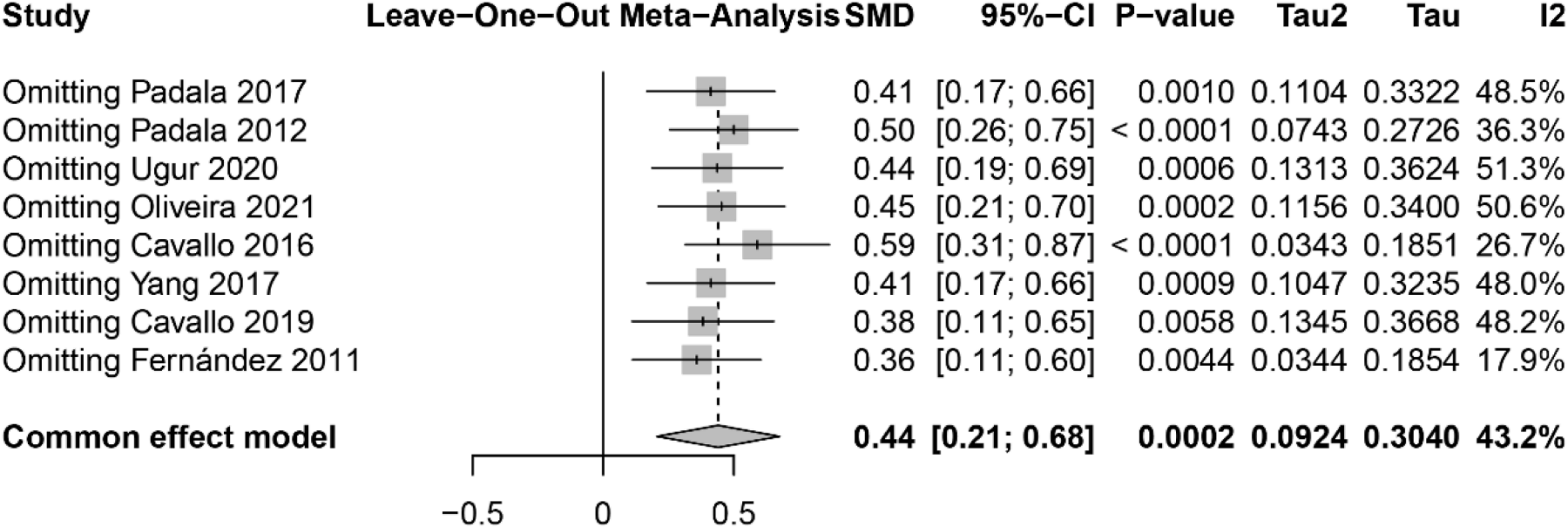
Sensitivity analysis of effect on global cognitive function in patients with AD.

Eight studies evaluated the effects of VR intervention on global cognitive function in patients with AD. The heterogeneity was moderate (I² = 43.2%), and a fixed-effects model was used. Participants receiving VR-based training demonstrated significantly better global cognitive function compared with controls (SMD = 0.44, 95%CI = 0.21 to 0.68, k = 8) (Fig. 3). Sensitivity analysis indicated that omitting any single study did not substantially alter the overall results (Appendix Fig. A2).

In subgroup analysis, four trials used VR-based exercise interventions and four used VR-based cognitive interventions. Heterogeneity was lower in the exercise subgroup (I² = 5.2%) than in the cognitive subgroup (I² = 65.6%). However, neither subgroup showed statistically significant effects (all p > 0.05). Similarly, no significant difference was observed between intervention programs with fewer than 24 sessions and those with 24 or more sessions (p > 0.05) (Table 2).

**Table 2:** Effects of VR-based training in patients with AD (N=11).

**Indicators**	**Global cognitive function**		**Executive function**		**Short-term memory**		**Spatial memory**		**Living ability**		**Live quality**		**Balance and coordination**		**Falling risk**		**Fear of Falling**		**Depression**	
Effects of VR intervention	SMD (95% CI)	k	SMD (95% CI)	k	SMD (95% CI)	k	SMD (95% CI)	k	SMD (95% CI)	k	SMD (95% CI)	k	SMD (95% CI)	k	SMD (95% CI)	k	SMD (95% CI)	k	SMD (95% CI)	k
**Main analysis**	**0.44(0.21,0.68)**	8	0.93(-0.23,2.09)	4	**0.62(0.25,0.99)**	**3**	0.92(-1.28,3.12)	3	-0.38(-1.6,0.84)	4	-0.25(-2.57,2.07)	3	1.08(-1.25,3.4)	5	-0.34(-0.88,0.21)	2	1.84(-12.95,16.63)	2	-0.82(-3.03,1.39)	3
**Subgroup analysis**																				
**(1) Type of intervention**																				
Cognitive intervention	0.63(-0.22,1.47)	4	1.16(-0.53,2.85)	3	**0.62(0.25,0.99)**	**3**	0.92(-1.28,3.12)	3	-1.31(-2.11, -0.51)	1	-1.36(-2.47, -0.24)	1	0.19(-0.69,1.07)	1	-	-	-	-	-0.82(-3.03,1.39)	3
Exercise intervention	0.33(-0.08,0.74)	4	0.19(-0.78,1.16)	1	-	0	-	0	-0.04(-0.53,0.45)	3	0.22(-0.36,0.81)	2	1.33(-2.03,4.69)	4	-0.34(-0.88,0.21)	2	1.84(-12.95,16.63)	2	-	-
**(2) Sessions**																				
<24	0.39(-0.18,0.96)	2	0.32(-0.33,0.98)	2	0.38(-0.51,1.26)	1	0.73(-0.18,1.64)	1	-0.04(-0.92,0.84)	1	0.22(-0.36,0.81)	2	**0.62(0.12,1.13)**	2	-0.66(-1.38,0.05)	1	**0.73(0.01,1.45)**	1	-0.69(-1.71,0.32)	1
≥24	0.52(-0.06,1.10)	6	**1.6(1.13,2.06)**	2	0.67(0.26,1.08)	2	0.98(-9.94,11.9)	2	-0.48(-2.7,1.73)	3	-1.36(-2.47, -0.24)	1	1.47(-5.11,8.05)	3	0.11(-0.72,0.95)	1	**3.06(1.82,4.3)**	1	-0.87(-11.95,10.2)	2
**(3) Disease severity**																				
Mild dementia	**0.35(0.08,0.62)**	5	1.16(-0.53,2.82)	3	**0.62(0.25,0.99)**	3	0.92(-1.28,3.12)	3	-0.06(-6.71,6.59)	2	0.22(-0.36,0.81)	2	1.47(-5.11,8.05)	3	0.11(-0.72,0.95)	1	**3.06(1.82,4.3)**	1	0.00(-0.88,0.88)	1
Moderate dementia	**0.70(0.24,1.16)**	3	0.19(-0.78,1.16)	1	-	0	-	0	-0.69(-8.76,7.38)	2	-1.36(-2.47, -0.24)	1	**0.62(0.12,1.13)**	2	-0.66(-1.38,0.05)	1	**0.73(0.01,1.45)**	1	-1.26(-7.9,5.39)	2
**(4) Type of control**																				
Conventional cognitive	**0.46(0.16,0.76)**	3	1.15(-7.25,9.54)	2	**0.57(0.16,0.98)**	2	1.33(-5.42,8.09)	2	-1.31(-2.11, -0.51)	1	-1.36(-2.47, -0.24)	1	-	-	-	-	-	-	-1.26(-7.9,5.39)	2
Conventional exercise	0.26(-6.3,6.82)	2	-	0	-	-	-	0	-0.06(-6.71,6.59)	2	0.22(-0.36,0.81)	2	2.17(-28.5,32.83)	2	0.11(-0.72,0.95)	1	**0.73(0.01,1.45)**	1	-	-
Routine medical	**0.51(0.03,1)**	3	0.65(-0.03,1.32)	2	0.87(-0.06,1.79)	1	0.08(-0.79,0.96)	1	-0.04(-0.92,0.84)	1	-	-	0.52(0.08,0.95)	3	-0.66(-1.38,0.05)	1	**3.06(1.82,4.3)**	1	0.00(-0.88,0.88)	1

An increase in score indicated better cognitive functions in global cognitive function, executive function, short-term memory, spatial memory, activities of daily living, quality of life, balance and coordination, fear of falling, risk of falling, and depression.

**VR,** virtual reality; **SMD,** standardized mean difference; **CI,** confidence interval; **k,** number of cohorts.

The effect sizes with statistical significance (at 95% confidence level) are highlighted in bold.

The effect size of VR training was larger in participants with moderate dementia (SMD = 0.7, 95%CI = 0.24 to 1.16, k = 3) than in those with mild dementia (SMD = 0.35, 95%CI = 0.08 to 0.62, k = 5), though this difference was not statistically significant (p > 0.05). Additionally, the effect size of VR-based training within the routine medical group (SMD = 0.51, 95%CI = 0.03 to 1, k = 3) was superior to that of both the conventional cognitive group (SMD = 0.46, 95%CI = 0.16 to 0.76, k = 3) and the conventional exercise group (SMD = 0.26, 95%CI = -6.3 to 6.82, k = 2), but the difference is not significant (p > 0.05) (Table 2).

### 3.5 Executive function in people with AD

**Fig. A3:**
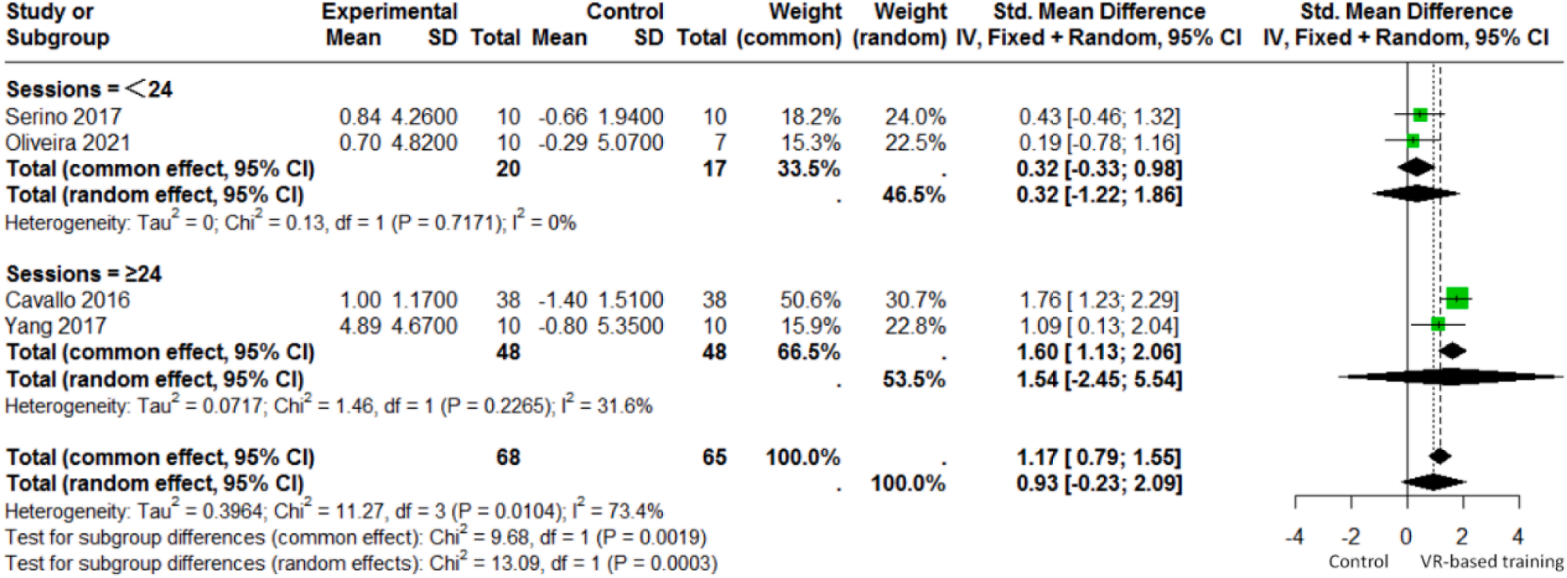
Effect of VR-based training on executive function in patients with AD.

Four studies evaluated the effects of VR intervention on executive function in individuals with AD. Due to substantial heterogeneity (I² = 73.4%), a random-effects model was applied. The pooled results indicated that VR intervention did not yield a statistically significant improvement in executive function compared to control conditions (SMD = 0.93, 95% CI = -0.23 to 2.09, k = 4) (Appendix Fig. A3).

Subgroup analysis based on intervention dose revealed that participants who received a higher number of sessions (≥24) showed significantly better executive function compared to controls (SMD = 1.6, 95% CI = 1.13 to 2.06, k = 2). In contrast, among those receiving fewer than 24 sessions, no significant difference was observed between the intervention and control groups (SMD = 0.32, 95% CI = -0.33 to 0.98, k = 2) (Table 2).

### 3.6 Memory in people with AD

**Fig. A4:**
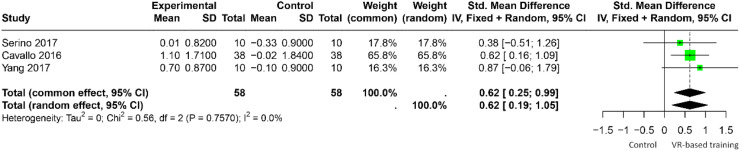
Effect of VR-based training on short-term memory in patients with AD.

Data on both short-term memory and spatial memory were extracted and analyzed. Three studies evaluated the effect of VR intervention on short-term memory in individuals with AD. Given minimal heterogeneity (I² = 0.0%), a common-effects model was employed. The results demonstrated that participants receiving VR intervention showed significantly greater improvement in short-term memory compared to the control group (SMD = 0.62, 95% CI = 0.25 to 0.99, k = 3) (Appendix Fig. A4).

Spatial memory outcomes were evaluated in three studies of VR interventions for AD patients. Substantial heterogeneity (I² = 83.5%) necessitated the use of a random-effects model, which indicated no statistically significant difference between the VR intervention and control groups (SMD = 0.92, 95% CI = -1.28 to 3.12, k = 3) (Table 2).

### 3.7 Living conditions of people with AD

To evaluate the living conditions of individuals with AD, outcomes related to the capacity for daily living and quality of life were analyzed. Four studies investigated the impact of VR intervention on the living abilities of those affected by AD. A considerable degree of heterogeneity was observed (I² = 70.3%), prompting the use of a random-effects model. The results showed no statistically significant improvement in living abilities among participants receiving VR intervention compared to controls (SMD = -0.38, 95% CI = -1.6 to 0.84, k = 4). Subgroup analysis similarly revealed no significant effects (p > 0.05).

Three studies assessed the impact of VR interventions on quality of life. The heterogeneity remained substantial (I² = 71.6%), which necessitated a random-effects model. The meta-analysis indicated no significant difference between the VR and control groups (SMD = -0.25, 95% CI = -2.57 to 2.07, k = 3). Further subgroup analysis also did not yield any significant results (p > 0.05).

### 3.8 Balance and coordination impairments in people with AD

Five investigations assessed the impact of VR interventions on balance and coordination deficits in individuals with AD. The heterogeneity was considerable (I² = 85%), necessitating the application of a random-effects model. Those who participated in the VR interventions exhibited effects that were not statistically significant (SMD = 1.08, 95% CI = -1.25 to 3.4, k = 5).

### 3.9 Fall risk and fear of falling in people with AD

Two studies evaluated the effects of VR intervention on fall risk in people with AD. With low heterogeneity observed (I² = 48.1%), a common-effects model was applied. The results indicated no statistically significant reduction in fall risk following VR intervention (SMD = -0.34, 95% CI = -0.88 to 0.21, k = 2). Subgroup analysis similarly showed no significant effects (p > 0.05).

Two studies assessed the impact of VR intervention on fear of falling. Due to high heterogeneity (I² = 90.2%), a random-effects model was used. Although individual studies suggested a potential benefit of VR intervention, the pooled effect size did not reach statistical significance (SMD = 1.84, 95% CI = -12.95 to 16.63, k = 2). Subgroup analysis revealed substantial methodological variations across trials in terms of intervention sessions, patient severity, and control interventions, which may have contributed to the observed heterogeneity (p < 0.01).

### 3.10 Depression in people with AD

Three studies evaluated the effects of VR intervention on depressive symptoms in individuals with AD. Given considerable heterogeneity among these studies (I² = 74.6%), a random-effects model was applied. The meta-analysis indicated no statistically significant improvement in depressive symptoms following VR intervention compared to control conditions (SMD = -0.82, 95% CI = -3.03 to 1.39, k = 3). Subgroup analysis further confirmed the absence of significant effects across different intervention protocols or patient characteristics (p > 0.05).

## 4 Discussion

This systematic review and meta-analysis examined the efficacy of VR-based training in patients with AD across cognitive, functional, and psychological domains. The synthesized results indicate that VR interventions can lead to clinically meaningful improvements in specific outcome measures, particularly global cognitive function and short-term memory. These findings suggest that immersive, technology-enhanced VR training modalities can effectively engage patients with AD and hold significant promise for enhancing targeted functional outcomes [38].

The included studies implemented VR interventions over durations of 4 to 12 weeks, with session frequencies ranging from 2 to 5 times per week and a maximum session length of 45 minutes. No adverse events were reported, suggesting that VR training within these parameters is safe and well-tolerated. This aligns with existing recommendations that patients with MCI or dementia engage in exergaming approximately 2 to 4 times per week [39]. The majority of protocols (approximately 82%) implemented interventions 2 to 3 times weekly, supporting both the feasibility and utility of VR training in individuals with AD.

All participants in this analysis had clinically diagnosed AD dementia, classified as mild or moderate based on MMSE scores. Notably, there remains a scarcity of VR studies focusing specifically on the MCI stage due to AD. Many existing trials involving MCI cohorts include mixed etiologies, such as vascular or frontotemporal causes, without a clear pathological distinction [[40], [41], [42], [43]]. This diagnostic heterogeneity complicates the interpretation of VR efficacy in early AD.

The widespread adoption of biomarker-based diagnostic frameworks, such as the ATN system (amyloid, tau, neurodegeneration) highlighted in the 2024 revised criteria [44], now enables more precise etiological stratification. Future VR trials should prioritize enrolling participants with biomarker-confirmed AD pathology to better assess its potential in the prodromal stage.

Pooled results from this meta-analysis demonstrated that VR-based training leads to significant improvements in global cognitive function among patients with AD, consistent with prior research in neurologic rehabilitation [45,46]. VR can enhance patient motivation and adherence by integrating cognitive and motor tasks within an engaging and ecologically valid setting [[47], [48], [49]]. Subgroup analyses further indicated that VR-based interventions yielded superior outcomes compared to conventional exercise, cognitive training, or usual care alone. These findings are in line with studies reporting a unique therapeutic benefit of VR over standard approaches [[50], [51], [52]].

Interestingly, we found that VR intervention appeared to be more effective in patients with moderate dementia than in those with mild dementia, although the difference was not statistically significant. Previous studies have suggested that the efficacy of VR intervention may be related to the characteristics of disease progression and the implementation features of VR [53]. Therefore, further research on VR interventions for AD patients at different disease stages deserves attention, so as to provide evidence for the precise formulation of non-pharmacological prescriptions.

A significant positive effect was also observed in short-term memory, corroborating findings from other VR studies [54,55]. Previous research suggests that physical exercise interventions may preferentially improve memory, whereas cognitive training tends to target executive functions [56,57]. In the present analysis, however, VR-based interventions did not yield statistically significant effects on executive functioning. Similarly, no significant benefits were detected in several other domains, including spatial memory, activities of daily living, quality of life, balance and coordination, fall risk, fear of falling, and depression. The absence of significant effects in these areas may reflect limited statistical power due to small sample sizes in the primary studies, as well as methodological variations in intervention protocols and outcome measurements.

The positive findings regarding global cognition and short-term memory, while encouraging, must be interpreted with caution. The overall evidence base remains nascent, characterized by a small number of trials with limited sample sizes. Consequently, the absence of statistically significant improvements in other critical domains is plausibly attributable to the​ insufficient statistical power​ in the primary studies rather than conclusive evidence of ineffectiveness. This pattern of mixed outcomes is consistent with broader reviews of VR interventions in older adults with cognitive impairment, underscoring the variability and preliminary nature of the current evidence [22,58]. Therefore, the positive effects observed here should be considered a signal of potential efficacy that requires confirmation in more robust studies.

The observed variations in effect sizes across domains, as well as the results of our subgroup analyses, further underscore the methodological heterogeneity inherent in the current VR literature for AD. Factors such as the degree of immersion, the specific design of the VR program, intervention dosage, and participant characteristics are recognized as potential moderators [19]. Our subgroup analyses (e.g., VR-cognitive vs. VR-exercise) attempted to disentangle these influences, but the limited number of studies within each subgroup precludes definitive conclusions. This highlights the need for future trials to consistently report detailed VR parameters to facilitate clearer interpretation and synthesis [59].

## 5 Conclusion

In summary, this meta-analysis provides preliminary evidence that VR-based training may have beneficial effects on global cognitive function and short-term memory in patients with AD. However, these findings are tempered by the limited number of studies, small sample sizes, and considerable methodological heterogeneity among the included trials. The lack of significant effects in other important functional and psychological domains likely indicates inadequate statistical power in the existing research rather than definitive ineffectiveness. Consequently, the current evidence remains insufficient to draw firm conclusions. Future large-scale, well-controlled RCTs focusing specifically on AD populations are essential to validate these potential benefits and establish evidence-based rehabilitation protocols.

## 6 Limitation

This research synthesized current evidence to assess virtual reality-based interventions on diverse cognitive and behavioral domains in AD cohorts, though methodological constraints warrant acknowledgment. First, the number of studies measuring global cognitive function and related outcomes was below 10, potentially compromising the robustness of pooled estimates. Subgroup analyses, which could help refine future study protocols, were constrained by limited sample sizes and inadequate statistical power. Second, most studies did not report intensity parameters for VR-based exercise interventions. Variability in intensity levels may influence therapeutic efficacy, and the lack of such data precludes further dose-response analysis. Third, participants were recruited from diverse settings (e.g., communities, hospitals, rehabilitation centers), and interventions varied widely in duration, frequency, and total sessions, introducing clinical and methodological heterogeneity that may affect the consistency of the results. Fourth, although several ongoing trials registered on ClinicalTrials.gov met the inclusion criteria, they were either not yet completed or had not released outcome data at the time of analysis. Consequently, this review relied solely on publicly available published data, which may be subject to publication bias.

## 7 Future research directions

To translate these preliminary findings into clinically actionable evidence and address the limitations of the current literature, future research should prioritize several key avenues. First, there is a clear need for large-scale, rigorously designed RCTs that are adequately powered to detect clinically meaningful effects. These trials should exclusively enroll participants with biomarker-confirmed AD pathology to ensure diagnostic specificity and enhance the generalizability of results to the target AD population. Second, achieving meaningful synthesis and comparison across studies requires the standardized reporting of VR intervention parameters. Future publications should consistently detail the level of immersion (fully immersive, semi-immersive, or non-immersive), specific hardware and software used, the description of cognitive or motor tasks, exercise intensity (for exergames), and the rationale for dosage (session duration, frequency, and total program length). Third, comparative effectiveness research is warranted to identify the active components of VR interventions. Head-to-head trials comparing immersive VR with non-immersive computerized cognitive training, traditional exercise, or usual care would clarify the added value of immersion and technological complexity. Finally, longitudinal studies are needed to determine the sustainability of any cognitive or functional benefits and to explore the potential of VR-based training to modify the trajectory of decline in AD.

## Fundings

This systematic review and meta-analysis is supported by grants from the National Natural Science Foundation of China (82471439) to G.C., and Hubei Provincial Natural Science Foundation of China (JCZRQN202501239) to J.W.

## Declaration of the use of generative AI and AI-assisted technologies in scientific writing and in figures, images and artwork

We have not used AI in the writing process or in the creation of figures.

## Ethical statement

Not required; analysis of aggregated identified clinical trial data.

## Data statement

The data that support the findings of this study are available from the corresponding author (Guiqin Chen) upon reasonable request.

## CRediT authorship contribution statement

**Junjie Wang:** Writing – original draft, Visualization, Validation, Software, Resources, Project administration, Methodology, Investigation, Funding acquisition, Formal analysis, Data curation, Conceptualization. **Can Wu:** Software, Investigation, Formal analysis, Data curation. **Kedong Zhu:** Visualization, Software, Investigation, Data curation. **Xiaoshan Qi:** Software, Investigation, Formal analysis, Data curation. **Guiqin Chen:** Writing – review & editing, Writing – original draft, Visualization, Validation, Supervision, Resources, Project administration, Investigation, Funding acquisition, Conceptualization.

## Declaration of competing interest

The authors declare no conflicts of interest.

## Appendix

Fig. A1, A2, A3, A4
